# Marine Diterpenoids as Potential Anti-Inflammatory Agents

**DOI:** 10.1155/2015/263543

**Published:** 2015-10-11

**Authors:** Yisett González, Daniel Torres-Mendoza, Gillian E. Jones, Patricia L. Fernandez

**Affiliations:** ^1^Centro de Biología Celular y Molecular de Enfermedades, Instituto de Investigaciones Científicas y Servicios de Alta Tecnología (INDICASAT AIP), Ciudad de Panamá, Panama; ^2^Department of Biotechnology, Acharya Nagarjuna University, Guntur 522510, India; ^3^Centro de Descubrimiento de Drogas y Biodiversidad, INDICASAT AIP, Ciudad de Panamá, Panama

## Abstract

The inflammatory response is a highly regulated process, and its dysregulation can lead to the establishment of chronic inflammation and, in some cases, to death. Inflammation is the cause of several diseases, including rheumatoid arthritis, inflammatory bowel diseases, multiple sclerosis, and asthma. The search for agents inhibiting inflammation is a great challenge as the inflammatory response plays an important role in the defense of the host to infections. Marine invertebrates are exceptional sources of new natural products, and among those diterpenoids secondary metabolites exhibit notable anti-inflammatory properties. Novel anti-inflammatory diterpenoids, exclusively produced by marine organisms, have been identified and synthetic molecules based on those structures have been obtained. The anti-inflammatory activity of marine diterpenoids has been attributed to the inhibition of Nuclear Factor-*κ*B activation and to the modulation of arachidonic acid metabolism. However, more research is necessary to describe the mechanisms of action of these secondary metabolites. This review is a compilation of marine diterpenoids, mainly isolated from corals, which have been described as potential anti-inflammatory molecules.

## 1. Introduction

Inflammation is a complex biological response against pathogens or tissue damage characterized by vasodilation, increased blood flow, vascular permeability, and cellular extravasation [1]. Macrophages, mast cells, and dendritic cells, resident in the tissues, are the first cells of innate immunity that detect and recognize the pathogen and initiate the inflammatory response [1]. Acute inflammation is an early response in which innate immune cells such as polymorphonuclear cells and monocytes are recruited to the site of irritation and secrete inflammatory mediators (e.g., cytokines, chemokines, and free radicals), which amplify the response [2]. Chronic inflammation, in turn, is the long-term inflammatory process that occurs as a dysregulation of acute inflammation often due to extended exposure to the initial irritant, persistent injury, or autoimmune disease. Chronic inflammation is associated with many pathological diseases including cancer, autoimmune diseases, atherosclerosis, rheumatoid arthritis, asthma, and cardiovascular diseases [3–5].

The search for new anti-inflammatory agents is challenging due to the complexity of the inflammatory process and its role in host defense. However, the progress attained in understanding the mechanisms involved in inflammation has made the identification of new targets possible, opening the range of search for new compounds with potential therapeutic effects on acute or chronic inflammatory diseases. Several drug discovery and development programs are focused on the search for bioactive compounds obtained from natural sources. Many drugs used today for the treatment of several diseases have been developed from natural products. The studies in terrestrial organisms have been extended to the marine environment, a resource with an enormous potential for drug discovery [6–8].

In the world of natural products, terpenoids are one of the largest and most studied groups of molecules. Terpenoids are secondary metabolites containing a C5 isoprene unit derived from a biosynthetic pathway based on mevalonate, which is essential for diverse cellular functions [9]. Terpenoids can be classified into hemi, mono, sesqui, di, sester, or tri based on the number of isoprene C5 units. These compounds are found largely in higher plants, but also in lower invertebrates including marine organisms.

Diterpenoids, in particular, are a promising class of molecules of secondary metabolites with a range of activity including antiviral, antibacterial, antiparasite, anticancer, and anti-inflammatory [10]. Diterpenes are comprised of four isoprene units with the chemical structure C_20_H_32_. Several studies have demonstrated a variety of diterpenoid structures presenting anti-inflammatory capacity. This review discusses the potential anti-inflammatory role of several diterpenoids derived from marine organisms.

## 2. Current Anti-Inflammatory Drugs

There are several classes of anti-inflammatory drugs available today, including nonsteroidal anti-inflammatory drugs (NSAIDs), glucocorticoids, and immunomodulatory drugs. NSAIDs, including aspirin, ibuprofen, and naproxen, are a widely administered class of drugs used for anti-inflammatory and analgesic purposes. Drugs in the NSAID category differ significantly in structure, but all share common mechanisms of action. These drugs prevent the release of prostaglandins through the inhibition of cyclooxygenase (COX) by covalently modifying the enzyme or by competing with the substrate for the active site [11, 12]. Side effects of these engineered drugs, however, are often severe and range from gastric ulcers to kidney damage and death.

Two COX isozymes are encoded in the human genome, COX-1 and COX-2. COX-1 is expressed in nearly all organs and cells but is most prominent in the stomach and in platelets, whereas COX-2 is an inducible, inflammation-specific isoform and regulates the synthesis of prostaglandins during inflammation [13–15]. Prostaglandins modulate different immune cell types including macrophages, dendritic cells, and T and B lymphocytes, leading to pro- and anti-inflammatory effects. Prostaglandins have several functions, including augmenting the blood flow and vascular permeability, regulating the expression of cytokines by innate immune cells, and inducing the expression of costimulatory molecules [16].

When it was discovered that the gross reduction or elimination of prostaglandins achieved by nonselective inhibition of COX enzymes often resulted in gastric ulcers [17, 18], researchers sought drugs that selectively inhibited COX-2. The resulting set of COX-2 selective drugs are collectively called coxibs and exhibit much lower rates of gastric ulcers than nonselective COX inhibitors [19, 20]. Unfortunately, it was then discovered that many coxibs also carry a higher risk of cardiovascular events such as coronary heart disease, heart attack, and stroke [21].

Glucocorticoids (GCs) are steroidal hormones that are naturally produced by vertebrates and function to control inflammation [22]. Because of their native role, synthetic GCs have been produced and used to treat a variety of inflammatory-related diseases including asthma [23], inflammatory bowel disease [24], rheumatoid arthritis [25], and systemic lupus erythematous [26]. Both synthetic and natural GCs act by binding to and activating the glucocorticoid receptor (GR), a transcription factor that acts as an activator or repressor of several genes by direct binding with specific DNA sequences or by interfering with the transcriptional activity of other transcription factors [27]. Several mechanisms have been proposed to explain the inhibitory effect of GCs on the transcription of inflammatory genes. Many of them are related to the inhibition of Nuclear Factor-*κ*B (NF*κ*B) activation at different levels, including a direct physical association of GR with NF*κ*B and the induction of the expression of the regulatory protein I*κ*B*α* [28].

NF*κ*B is a constitutively expressed protein present in nearly all cell types. It has been implicated in the regulation of apoptosis genes, cell adhesion molecules, stress responses, cancer, immune system, and inflammatory responses [29, 30]. In inflammation, NF*κ*B regulates the transcription of inflammatory genes induced by a variety of intra- and extracellular stimuli. The activation of NF*κ*B and its translocation to the nucleus depends on the phosphorylation and degradation of the I*κ*B proteins [31, 32]. It has been shown that several NSAIDs also inhibit NF*κ*B activation independently of their effect on COX inhibition [33–36]. These agents include aspirin, salicylates, sulindac, and sulphasalazine. However, until now inhibitors of NF*κ*B with a comparable anti-inflammatory capacity as glucocorticoids have not been identified. Although glucocorticoids, like dexamethasone, prednisone, and hydrocortisone, are successful at treating many inflammatory based diseases, continued use may lead to adverse events such as bruising, cataracts, muscle weakness, skin changes, sleep disturbances, weight gain, or more severe side effects such as type II diabetes mellitus, osteoporosis, and psychiatric symptoms [37–39].

Immunomodulatory drugs, such as thalidomide and its analogs, are also inhibitors of NF*κ*B activation [40]. These drugs have anticancer, anti-inflammatory, and antiangiogenic actions by modulating the secretion of cytokines such as Tumor Necrosis Factor Alpha (TNF-*α*) and interleukins IL-6 and IL-12 [41–43]. It has been proposed that the anti-inflammatory effect of these drugs occurs by inhibition of I*κ*B degradation and downregulation of NF*κ*B DNA-binding activity. Inhibitors of TNF-*α* are also being used currently for the treatment of inflammatory diseases such as rheumatoid arthritis, Crohn's disease, and asthma [44]. These molecules act by inhibiting the binding of TNF-*α* to its receptor or by neutralizing the soluble and the membrane-bound forms of TNF-*α* [44, 45]. However, several adverse effects for these drugs have been described, such as heart failure, increased predisposition to infection, and exacerbation of latent tuberculosis [44, 46].

There are many natural remedies for inflammation and pain, such as curcumin and green tea, which act via similar mechanisms but exhibit limited, if any, unwanted side effects [47]. Curcumin, a compound found in turmeric, has also been described to confer anti-inflammatory effects through a combination of mechanisms including inhibition of COX-2, lipoxygenase, and the NF*κ*B pathway [48–51]. In addition to anti-inflammatory effects, curcumin has also been attributed with antitumor [52, 53], antiviral [54], and antibacterial [55] effects. Curcumin is being tested for efficacy in patients with ulcerative colitis [56–58].

Epigallocatechin-3-gallate (EGCG) is the main component in green tea that is responsible for conferring not only anti-inflammatory effects but also antiviral [59, 60], antibacterial [61], and anticancer effects [62, 63]. The anti-inflammatory effects are achieved most notably through COX-2 inhibition at the RNA and protein level [64]. Interestingly, EGCG has not been found to have an effect on COX-1 expression.

## 3. Marine-Derived Diterpenoids as Anti-Inflammatory Compounds

De las Heras and Hortelano in 2009 compiled a comprehensive list of the most promising anti-inflammatory diterpenoids, almost all of which were extracted from plants [10]. In their compilation, they describe the mechanisms of action associated with inhibition of the NF*κ*B signaling pathway of most families of diterpenoids. Bioactive diterpenoids act in the NF*κ*B pathway by blocking a range of activities including DNA-binding, IKK complex activation, and I*κ*B phosphorylation. Clinical studies have shown that commercial extracts from medicinal plants that contain large concentrations of diterpenoids that inhibit the NF*κ*B pathway are effective in reducing symptoms of rheumatoid arthritis [65, 66]. These extracts have also been tested in the treatment of other autoimmune and inflammatory diseases showing efficacy with variable mild side effects [67–69]. In this regard we discuss different families of diterpenoids isolated from marine organisms with anti-inflammatory capacity. Several of those molecules are promising candidates for further anti-inflammatory drug development.

### 3.1. Eunicellane Diterpenoids

Anti-inflammatory activity for eunicellin-based diterpenoids has been reported in the last few years. This class of compounds is secondary metabolites that present the cladiellane skeleton with a C2-C9 or C2-C6 oxygen bridge. Eunicellin-based diterpenes include krempfielins, hirsutalins, klymollins, klysimplexin, klysimplexin sulfoxide, simplexin, and cladieunicellin and have been isolated and identified from soft corals belonging to the genera* Cladiella* or* Klyxum*. Some of these compounds have shown the capacity to inhibit the upregulation of inducible nitric oxide synthases (iNOS), COX-2, or IL-6 proteins in RAW 246.7 macrophages stimulated with lipopolysaccharide (LPS) [70–79]. Other compounds of these types were identified as inhibitors of superoxide generation and elastase release by N-formyl-methionyl-leucyl-phenylalanine/cytochalasin B (FMLP/CB) induced human neutrophils [80–87]. The release of superoxide and elastase by immune cells, mainly neutrophils, is important for the killing of host invading microorganisms but also contributes to host tissue damage during a chronic inflammatory disease. The mechanisms of action by which these compounds exert their anti-inflammatory effect have not been elucidated yet. Interestingly, most of these compounds selectively influenced certain inflammatory responses without affecting others (Table 1 and Supplementary Material available online at http://dx.doi.org/10.1155/2015/263543).

**Figure 1:**
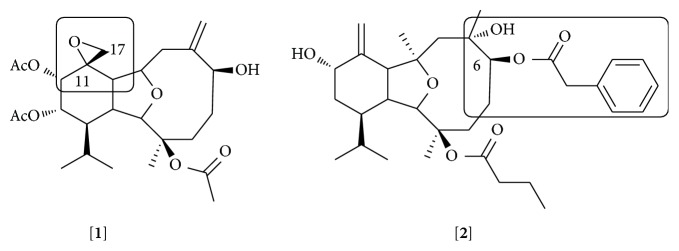
Structures of klymollin G [**1**] and klymollin M [**2**]. The epoxy group at C-17 for klymollin G and phenylacetate group at C-6 are labeled.

Reports have proposed that an epoxy group on C-11/C-17 present in some members of the klymollins is important for the inhibitory activity on iNOS expression [74]. However, some compounds of this family, the klymollins F and G (Figure 1, e.g.,** 1**), were significant inhibitors of both iNOS and COX-2, suggesting that the modulation of these enzymes might be due to the inhibition of a common molecule upstream in the signaling pathway that governs their expression. These two compounds, in addition to the epoxy, present a fatty acid residue at C-6 position attributing a micelle-like feature to the structure that might be important for membrane diffusion. Other authors have attributed the capacity of inhibiting elastase release and superoxide generation of klymollin M (**2**) to the presence of a phenylacetate group at C-6 (Figure 1) [83]. Comparing the structure of klymollin M with other eunicellin-based diterpenoid inhibitors of elastase release, it appears that the presence of a butyric acid at C-3, a common feature of these molecules, might be also important for this activity (Figure 1 and Supplementary Material). Further studies are necessary to identify the structural components that play a role in the anti-inflammatory effect of these molecules and to describe their mechanisms of action.

**Figure 2:**
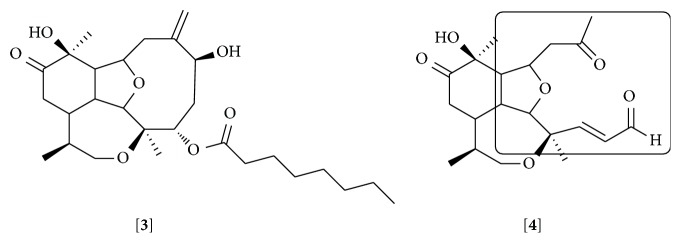
Structures of briarellin S [**3**] and* seco*-briarellinone [**4**]. The opening of the 10-member ring and the presence of carbonyl groups in the* seco*-briarellinone that have been suggested as being responsible for the higher anti-inflammatory effect than briarellin S are labeled.

Briarellins are another class of eunicellane diterpenoid. Most of the briarellins have been isolated from corals of the genera* Briareum* and* Pachyclavularia*. The anti-inflammatory activity of this family has been little explored. Our group recently showed that briarellin S (**3**) inhibits the production of nitric oxide (NO) by primary murine macrophages stimulated with LPS. This effect was smaller than the one observed when cells were exposed to LPS in the presence of* seco*-briarellinone (**4**). Differences in the IC_50_ of briarellin S (20.3 *μ*M) and* seco*-briarellinone (4.7 *μ*M) might be due to the opening of the 10-member ring and the presence of carbonyl groups in the* seco*-briarellinone, which is the main structural difference with the briarellin S [88] (Figure 2). The ester moiety present in the molecule of briarellin S could also be interfering with the activity of this compound. Structural modifications of these molecules would give a clue about the groups responsible for the anti-inflammatory effect.

### 3.2. Briarane Diterpenoids

Briarane diterpenoids form a family of compounds that present a basic chemical structure of a [8.4.0] bicycle carbon skeleton with most members containing a *γ*-lactone moiety. These compounds have been exclusively isolated from soft corals belonging to the order Gorgonacea (reviewed by [89]) and genera including* Briareum*,* Dichotella*,* Junceella*, and* Verrucella*. Around 600 briarane diterpenoids have been identified with a variety of bioactivities including antimicrobial, cytotoxic, and in some cases anti-inflammatory effects. Briaranes, such as frajunolides, juncenolides, and the briarenolides, are inhibitors of the superoxide generation and elastase released by human neutrophils stimulated with FMLP/CB [90–98].

Compounds isolated from* Junceella juncea*, the juncenolides, have shown moderate inhibition in the release of elastase [99] and junceol has presented weak inhibitory effects on neutrophil superoxide generation [100, 101]. However, neither the mechanisms of action nor the structural components involved in these differences in anti-inflammatory activity have been described. The inhibitory effect of briarane compounds on COX-2 and iNOS expression induced by LPS in macrophages has been also reported [102, 103].  Table 2 shows a compilation of briarane diterpenoids with anti-inflammatory properties.

**Figure 3:**
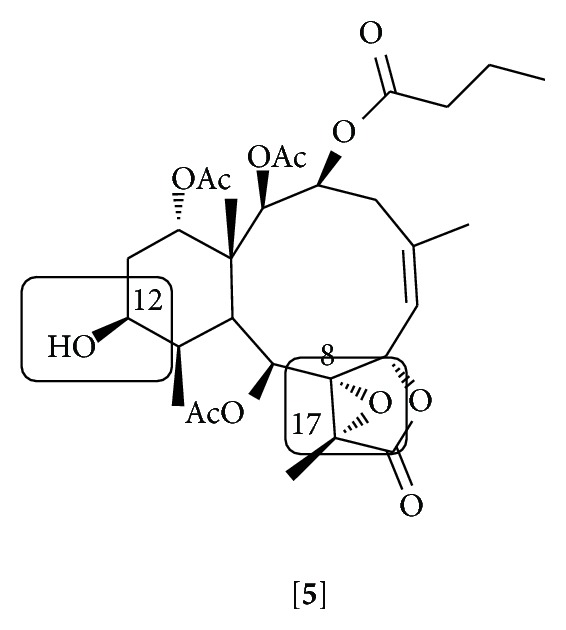
Structure of excavatolide B [**5**]. The 8,17-epoxide and 12-hydroxyl groups that have been suggested as being responsible for the anti-inflammatory effect are marked.

Excavatolide B (BrD1) (**5**), a briarane diterpenoid isolated from the coral* Briareum excavatum*, demonstrates* in vitro* and* in vivo* anti-inflammatory activity [104]. This compound inhibited vascular permeability and edema and decreased the expression of iNOS, COX-2, and matrix metallopeptidase (MMP-9) when topically applied in the skin of mice with 12-O-tetradecanoylphorbol-13-acetate- (TPA-) induced dermatitis. This effect might occur by a mechanism involving the inhibition of NF*κ*B and Akt activation observed in the skin of the animals. Comparing the effect on IL-6 secretion induced by LPS in bone marrow derived dendritic cells (BMDC) of different briarane diterpenoids isolated from the same coral and semisynthetic analogs of BrD1, the authors concluded that 8,17-epoxide and 12-hydroxyl groups are essential for the inhibition of IL-6 secretion by BrD1 [104] (Figure 3).

### 3.3. Cembrane Diterpenoids

Cembranes are a large family of diterpenoids isolated from terrestrial and marine organisms that exhibit a range of biological activities including antibacterial, antitumor, anti-inflammatory, and antiviral effects [105]. The basic structure of cembrane diterpenoids is constituted by a common 14-membered carbocyclic skeleton and usually presents cyclic ether, lactone, or furan moieties around this nucleus (reviewed by [106]). Unconventional cembranoids with 12-, 13-, or 14-membered variants have also been described [107, 108]. Cembranoids from marine organisms are mainly isolated from corals of the genera* Sinularia*,* Lobophytum, Eunicea*, and* Sarcophyton*.

**Figure 4:**
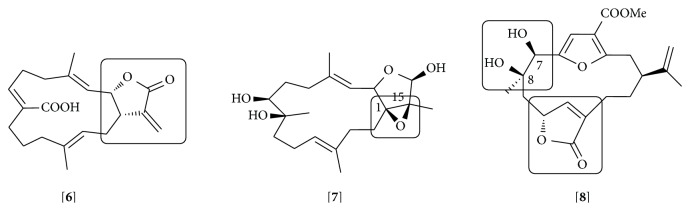
Cembrane diterpenoids: lobohedleolide [**6**], lobocrasol A [**7**], and sinumaximol B [**8**]. The presence of a *α*-methylene-*γ*-lactone in lobohedleolide (and cembranolides); an epoxy group at C-1/C-15 in lobocrasol A and a hydroxyl group at C-7 and/or C-8 in sinumaximol B are labeled.

Anti-inflammatory activity for different groups of cembrane diterpenoids has been reported. Cembranoids such as gibberosenes, grandilobatin, querciformolides, sarcocrassocolides, crassumolides, crassarines, sinularolides, durumolides, and columnariols have shown a capacity to inhibit the expression of iNOS and/or COX-2 by LPS-stimulated RAW 264.7 cells [109–121] (Table 3 and Supplementary Material). The presence of a *α*-methylene-*γ*-lactone in cembranolides has been suggested to be essential for the inhibition of iNOS expression [119] (Figure 4, e.g.,** 6**).

Some cembranoids have been identified as modulators of NF*κ*B signaling pathway [122–126]. Compounds from the crassumolide and laevigatol groups have shown dose-dependent inhibitory effects on the mRNA expression of iNOS and COX-2 induced by TNF-*α* in HepG2 cells by a mechanism that involved the inhibition of NF*κ*B transcriptional activation [123, 124]. The cembrane lobohedleolide (**6**) isolated from* Sarcophyton* sp. showed inhibitory activity on the production of TNF-*α* in LPS-stimulated RAW 264.7 cells [127]. This effect was later attributed to the ability of this compound to inhibit the degradation of I*κ*B*α* and the binding of NF*κ*B to the DNA [122]. However, lobohedleolide also induced an increase in the production of IL-8 in LPS-stimulated THP-1 cells through the activation of the IL-8 promoter region [122]. High levels of IL-8 have been found in some human cancers and have been associated with tumor progression and metastasis [128–130]. Thus, the identification of new anti-inflammatory molecules must be accompanied by a rigorous description of the mechanisms involved in the effect. Considering the pharmacological properties of lobohedleolide in the inhibition of NF*κ*B pathway, synthetic analogs could be produced with structural modifications that might favor the anti-inflammatory properties.

Members of the cembrane diterpenoids, lobocrasols isolated from* Lobophytum crassum*, have also shown inhibitory activity on NF*κ*B activation in TNF-*α* stimulated HepG2 with consequent decreases in COX-2 and iNOS gene expression [125]. The presence of an epoxy group at C-1/C-15 in the active compounds appears to be essential for the anti-inflammatory effect (Figure 4, e.g.,** 7**). Cembrane sinumaximols B and C isolated from* Sinularia maxima *were identified as potent inhibitors of IL-12 secretion by dendritic cells stimulated with LPS [131]. This activity could be attributed to the lactone moiety present in these molecules. Later, it was demonstrated that the sinumaximols A, B, and G inhibited the transcriptional activity of NF*κ*B induced by TNF-*α* in HepG2 cells and the expression of the intracellular adhesion molecule (ICAM-1) and iNOS [126]. Authors suggested that hydroxyl groups at C-7 and/or C-8 are responsible for the anti-inflammatory activity of these compounds. One of those compounds, sinumaximol B (**8**), exhibited inhibitory activity in both dendritic and HepG2 cells (Figure 4). It is important to note that only sinumaximol B contains the lactone and the hydroxyl at C-7 and C-8.

### 3.4. Diterpene Glycosides

Marine diterpene glycosides are derivatives exclusively produced by Gorgonian corals [132]. A diterpene aglycone core and a carbohydrate moiety characterize this class of compounds. Among the marine diterpenes glycosides, eleutherobins, fuscosides, and pseudopterosins are the most studied compounds [132]. The pseudopterosins (Ps) have been described as molecules with important anti-inflammatory and analgesic properties and were the first to be isolated from* Pseudopterogorgia elisabethae *[133, 134]. Pseudopterosin A (**9**) was identified as a potent anti-inflammatory agent, with a greater effect than the NSAID indomethacin, in the phorbol myristate acetate- (PMA-) induced topical inflammation animal model [133]. Pseudopterosin A also inhibited prostaglandin E_2_ and leukotriene C_4_ secretion in zymosan-stimulated murine peritoneal macrophages [135]. This molecule inhibited phagosome formation and triggered intracellular calcium release by a mechanism that involved its binding to a G protein coupled receptor [136]. Other pseudopterosins with exceptional anti-inflammatory activity also have been identified [137, 138] and are suggested to inhibit the synthesis of leukotrienes and the degranulation of human neutrophils [135, 137] (Table 4 and Supplementary Material). Several analogs of Ps such as* seco*-pseudopterosins and amphilectosins reduced the mouse ear edema induced by different inflammatory stimuli [138, 139] and the levels of myeloperoxidase at the inflammation site [139].

**Figure 5:**
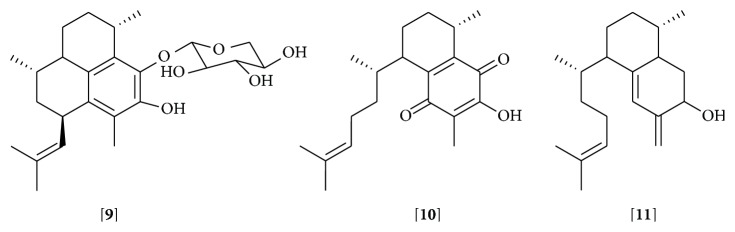
Pseudopterosin A [**9**], elisabethadione [**10**], and elisabethatrienol [**11**]. Glycoside diterpene, pseudopterosin A, nonglycoside diterpenes, elisabethadione, and elisabethatrienol.

Due to the relevant anti-inflammatory properties of Ps, they have attracted great attention from the organic chemistry community and new synthetic pseudopterosins have been obtained. Discussions of Ps syntheses are out of the scope of this review but they can be found elsewhere [reviewed by [132]]. It appears that the location and identity of carbohydrate moiety are not relevant for the anti-inflammatory activity; instead, the intact diterpene glycoside is needed for the Ps biological effect [140]. However, nonglycosylated compounds structurally related to the aglycone component of Ps, such as elisabethadione (**10**) and elisabethatrienol (**11**), have shown anti-inflammatory activity [138, 139] (Figure 5). Simplified structural analogs of the Ps and* seco*-Ps have been synthesized, which conserve the anti-inflammatory effect, suggesting that a more accessible aglycone would be sufficient for the activity [141, 142]. A semisynthetic derivative of pseudopterosin A maintaining the anti-inflammatory capacity has been obtained [143]. Due to their anti-inflammatory properties natural extracts from* P. elisabethae* rich in pseudopterosins are used in commercial skin care products [144].

Fuscosides have been isolated from the coral* Eunicea fusca*. Fuscosides A and B exhibit anti-inflammatory activity [145, 146]. Both compounds, when topically applied, reduce PMA-induced edema in mouse ears by inhibiting neutrophil infiltration. Fuscoside B inhibits the synthesis of leukotriene C_4_ in calcium ionophore-activated murine macrophages [145, 146]. It was demonstrated using cultures of human leukocytes that fuscoside B is a selective inhibitor of 5-lipoxygenase [147] (Table 4 and Supplementary Material). The aglycone precursor of fuscoside B, the fuscol, and other compounds as eunicol and the analogous eunicidiol, isolated from* E. fusca*, have also shown anti-inflammatory activity by reducing the edema induced by PMA in mouse ear [148]. Different approaches for the synthesis of naturally occurring fuscosides, conserving the anti-inflammatory capacity, have been attempted unsuccessfully.

Other members of the diterpene glycosides compounds have also shown anti-inflammatory activity. A calyculaglycoside isolated from* Eunicea* sp. exhibited topical anti-inflammatory activity in two* in vivo* assays, and it was suggested as a nonselective inhibitor of the 5-lipoxygenase and COX pathways [149]. It is relevant to note that compounds belonging to this family have the same aglycone (dilophol) and only differ in the identity of the carbohydrate moieties. Anti-inflammatory activity has not been reported for the eleutherobin compounds; however, two nonglycoside compounds, the valdivones A and B, which are related to the eleutherobin aglycone, inhibited chemically induced inflammation in mouse ear [150]. These findings question the relevance of carbohydrate moiety for the biological activity of glycoside compounds.

### 3.5. Other Diterpenoids

**Figure 6:**
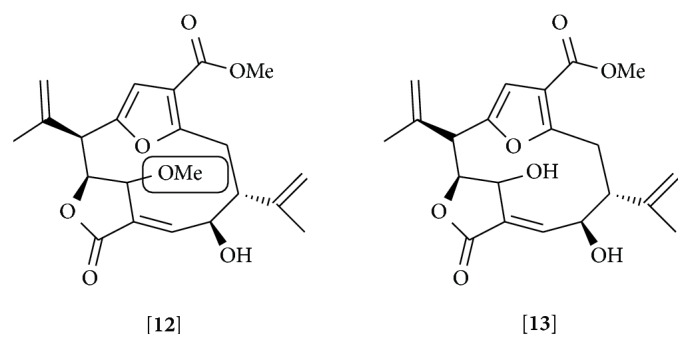
Pseudopterolide derivative [**12**] and isogorgiacerodiol [**13**]. The methoxyl group at C-9 in pseudopterolide derivative that has been suggested as being responsible for the higher anti-inflammatory effect than isogorgiacerodiol is labeled.

Pseudopteranes are only found in corals of the genera* Pseudopterogorgia*. Their ring system could be originated from a ring contraction reaction of a cembrane precursor [151]. Pseudopterolide 1 was the first compound identified and isolated from* Pseudopterogorgia acerosa* [152]. Other pseudopterane compounds include kallolides and isogorgiacerodiol isolated from* P. kallos* and* P. acerosa*, respectively [153, 154]. Pseudopterolide 1 and some kallolides have shown anti-inflammatory capacity in topical skin inflammation induced by PMA [152, 153]. Our group has recently demonstrated that pseudopterolide 1 derivative (**12**) also exhibits anti-inflammatory capacity. This compound inhibited the secretion and/or mRNA expression of a variety of inflammatory mediators (TNF-*α*, IL-6, NO, IP-10, iNOS, COX-2, and MCP-1) induced by TNF-*α* and ligands of TLRs in mouse peritoneal macrophages [155]. This effect was due to the capacity of this compound to inhibit I*κ*B*α* phosphorylation and the subsequent activation of NF*κ*B. The compound also inhibited the expression of macrophages activation markers such as CD80 and CD86 suggesting a role in the modulation of a variety of processes occurring during macrophage activation. The methoxyl group at C-9 appears to be important in the anti-inflammatory effect as it was more potent than the isogorgiacerodiol pseudopterane (**13**), which has a hydroxyl group at the same position [155] (Figure 6). It is notable how subtle structural differences in small molecules are essential for modifying their immune modulation activities.

Smaller groups of diterpenoids called verticillane-based and norditerpenoids, isolated from coral of the genera* Cespitularia* and* Sinularia*, respectively, have been recently identified to have anti-inflammatory capacity [156, 157]. It has been reported that members of these families, for example, cespitularin (verticillane-based diterpenoid), isolated from* C. hypotentaculata* and a series of norcembranolides, gyrosanolides, and other norditerpenoids isolated from* S. gyrosa*, inhibit the expression of iNOS in LPS-stimulated RAW 264.7 cells [156, 157].

The neorogioltriol, a tricyclic brominated diterpenoid isolated from the red algae* Laurencia glandulifera*, showed anti-inflammatory effects* in vitro* and* in vivo* [158]. This compound inhibited the activation of NF*κ*B and the production of TNF-*α*, COX-2, and NO in RAW 264.7 macrophages stimulated with LPS. The systemic administration of neorogioltriol reduced the edema formation in an animal model of carrageenan-induced local inflammation.

Dolabellane diterpenoids have been isolated mainly from plants but are also present in marine organisms. These compounds have a 5,11-bicyclic skeleton and exhibit antiviral, antiprotozoa, and antibacterial properties [159]. Recently, it has been suggested that the dolabelladienetriol, isolated from the brown marine alga* Dictyota pfaffii*, downregulates the production of TNF-*α* and NO through the inhibition of NF*κ*B activation in* Leishmania amazonensis* infected and uninfected macrophages, conferring an anti-inflammatory activity to this compound [160]. To our knowledge, this is the first report of anti-inflammatory capacity described for a marine-derived dolabellane diterpenoid.

## 4. Conclusions

Many efforts have been made to identify new anti-inflammatory molecules from natural sources. Terrestrial organisms are commonly used in traditional medicine to treat inflammatory diseases and have often been ascribed diterpenoid compounds to the anti-inflammatory effects. Marine invertebrates are exceptional sources of new molecules with therapeutic potential including diterpenoids secondary metabolites, which exhibit notable anti-inflammatory properties.

The anti-inflammatory capacity of some diterpenoids isolated from marine organisms is due to the inhibition of the NF*κ*B signaling pathway at different levels [122, 155]. NF*κ*B plays a crucial role in regulating the inflammatory responses and in the development of various human pathological conditions. Hence, this transcription factor constitutes a suitable target for the development of new anti-inflammatory drugs. Moreover, some marine diterpenoids have been shown to be inhibitors of prostaglandins and leukotrienes secretion and in some cases found to be selective inhibitors of 5-lipoxygenase and COX enzymes [147, 149]. Together, this evidence demonstrates that marine diterpenoids show a capacity of inhibiting different pathways involved in inflammation, supporting their potential for anti-inflammatory drugs development. However, little is known about the molecular mechanisms involved in the anti-inflammatory characteristics of marine diterpenoids. Thus, further studies are necessary to better understand their mechanisms of action.

The largest limitation for the study of natural products is the small amount of compounds that are obtained and the variations on their production that are influenced by the environmental changes to which marine organisms are exposed. Due to the potential applications of coral-derived compounds, coral aquaculture has been proposed as a way to establish a stable supply of bioactive materials for the extraction of natural products [161]. Some laboratories use this approach for the production of marine invertebrates with bioprospecting purposes. Importantly, natural growth rate of these organisms is not enough to sustain pharmaceutical exploitation. Many researchers have developed new strategies for the synthesis of compounds that conserve the biological activities of their natural analogs; nonetheless, it remains a challenging area.

## Supplementary Material

Supplementary Material contains the structures and names of all compounds with potential anti-inflammatory effect mentioned in the text and tables. Compounds are grouped by family as it is described in the article.

## Figures and Tables

**Table 1:** Anti-inflammatory effect of eunicellane diterpenoids.

	Families name	Biological source	COX-2	iNOS	Superoxide anion generation	Elastase release	Other
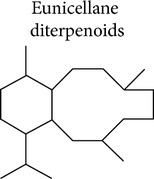	Krempfielins	*Cladiella krempfi *		B-C, D^*∗*^ [73] E^*∗*^, G, I [77]		K, M [82] N, P [84]	
	Hirsutalins	*C. hirsuta *	B [71]	B-D, H [71] K [78]		N [86] S [87]	
	Cladieunicellins	*Cladiella *sp.			C-E [80] 6-*epi* F [81]	A, C-D [80] 6-*epi* F [81]	
	Klymollins	*Klyxum molle *	F-G [74]	C-H [74]	M [83]	M [83]	X [79]
	Klysimplexins	*K. simplex *	R-S [72]	J-N, R-S [75]			
	Klysimplexin sulfoxides	*K. simplex *	C [72]	A-C [72]			
	Simplexin	*K. simplex *	E [70]	A, D-E [70]			

Data refer to compounds with percentage of inhibition > 50% for COX-2 and iNOS and >25% for superoxide anion generation and elastase release.

^*∗*^Percentage of inhibition 40–50%.

**Table 2:** Anti-inflammatory effect of briarane diterpenoids.

	Families name	Biological source	iNOS	Superoxide anion generation	Elastase release
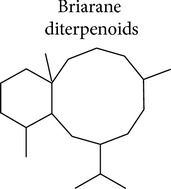	Frajunolide	*Junceella fragilis *		P-Q [97]	P-Q [97]
	Juncenolide	*J. juncea *		H [92] O [99]	N-O [99]
	Junceol	*J. juncea *		A-C [101] E [100]	
	Briarenolides	*Briareum* sp.	K-L [103]	F [95] I [96] J [98]	E [94] F [95] J [98]

Data refer to compounds with percentage of inhibition > 50% for iNOS and >25% superoxide anion generation and elastase release.

**Table 3:** Anti-inflammatory effect of cembrane diterpenoids.

	Families name	Biological source	COX-2	iNOS	NF*κ*B
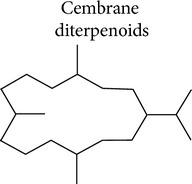	Crassarines	*Sinularia crassa *		H [118]	
	Grandilobatins	*S. grandilobata *		D [110]	
	Querciformolides	*S. querciformis *	C [111]	C [111] E [115]	
	Sinumaximols	*S. maxima *			A-C, G, I [126]
	Sarcocrassocolides	*Sarcophyton crassocaule *	I [117] Q [120]	A-D [116] F-L [117] M-O [119] P-R [120]	
	Crassocolides	*S. crassocaule *	A, E [120]	A-B, D-E [120]	
	Crassumolides	*Lobophytum crassum*	A, C [112]	A-C, F [112]	
	Crassumols	*L. crassum *			E [124]
	Lobocrasols	*L. crassum *			A-C [125]
	Durumolides	*L. durum *	C [113] F [114]	A-E [113] F-L [114]	
	Laevigatols	*L. laevigatum *			A-B [123]
	Columnariols	*Nephthea columnaris *(cultured coral)	A-B [121]	A-B [121]	

Data refer to compounds with percentage of inhibition > 50% for COX-2 and iNOS and IC_50_ values < 50 *μ*M in NF*κ*B.

**Table 4:** Glycosides diterpenes.

Families name	Biological source	Bioactive compounds
Pseudopterosins	*Pseudopterogorgia elisabethae *	Pseudopterosins A [133], E [137], Q [134], P, T, U [139]
Fuscosides	*Eunicea fusca *	Fuscosides A-B [145]
Eleutherobins	*Eunicea* sp.	Calyculaglycoside B [149]

Data refer to glycosides diterpenoids with anti-inflammatory activity.
