# Supplementary material for: Marine Diterpenoids as Potential Anti-Inflammatory Agents
Source: Mediators Inflamm. 2015 Oct 11;2015:263543. doi: 10.1155/2015/263543 (PMC4619941; doi:10.1155/2015/263543)
Supplement: Supplementary file 1 — Supplementary Material contains the structures and names of all compounds with potential anti-inflammatory effect mentioned in the text and tables. Compounds are grouped by family as it is described in the article. [file 263543.f1.pdf]

# ANTI-INFLAMMATORY MARINE EUNICELLANE DITERPENOIDS

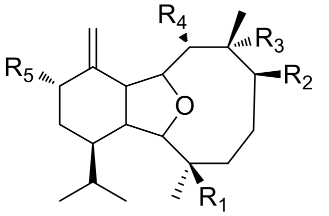


krempfielin B R$_1$ = OCO(CH$_2$)$_2$CH$_3$, R$_2$ = OMe, R$_3$ = OH, R$_4$ = OH, R$_5$ = H
krempfielin C R$_1$ = OCO(CH$_2$)$_2$CH$_3$, R$_2$ = OAc, R$_3$ = OH, R$_4$ = OH, R$_5$ = H
krempfielin D R$_1$ = OCO(CH$_2$)$_2$CH$_3$, R$_2$ = OMe, R$_3$ = OH, R$_4$ = OH, R$_5$ = OAc
krempfielin G R$_1$ = OCO(CH$_2$)$_2$CH$_3$, R$_2$ = =O, R$_3$ = H, R$_4$ = OH, R$_5$ = OAc
krempfielin I R$_1$ = OCO(CH$_2$)$_2$CH$_3$, R$_2$ = OAc, R$_3$ = OH, R$_4$ = OCO(CH$_2$)$_2$CH$_3$, R$_5$ = H
krempfielin M R$_1$ = OAc, R$_2$ = OH, R$_3$ = OH, R$_4$ = OH, R$_5$ = OAc
krempfielin N R$_1$ = OCO(CH$_2$)$_2$CH$_3$, R$_2$ = OMe, R$_3$ = OH, R$_4$ = H, R$_5$ = OH

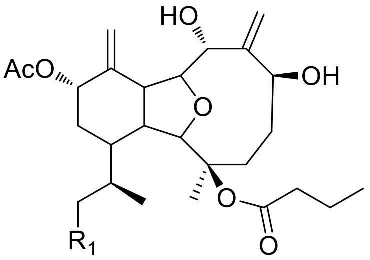


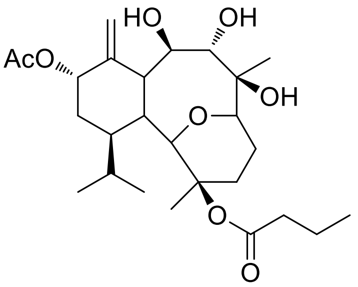


krempfielin E R$_1$ = OAc
krempfielin P R$_1$ = H

krempfielin K

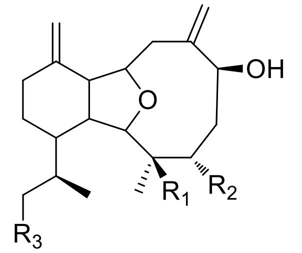


hirsutalin B R$_1$ = OH, R$_2$ = 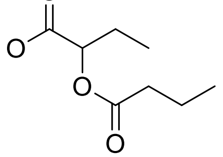 , R$_3$ = OAc

hirsutalin C R$_1$ = 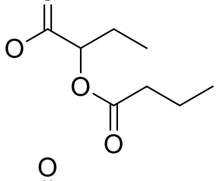 , R$_2$ = H, R$_3$ = OH

hirsutalin D R$_1$ = 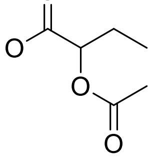 , R$_2$ = H, R$_3$ = OH

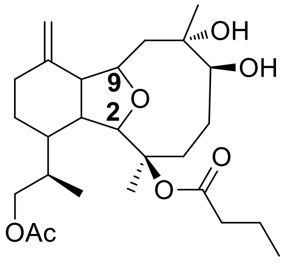

hirsutalin H

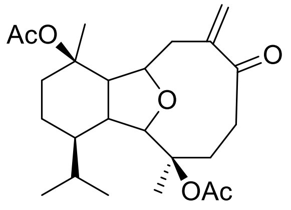

hirsutalin K

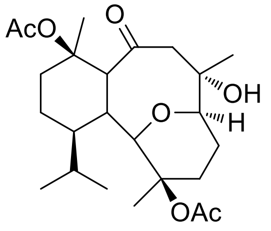

hirsutalin N

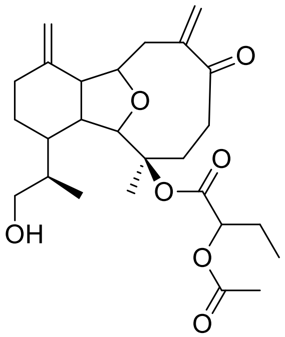

hirsutalin S

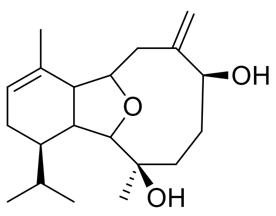

cladieunicellin A

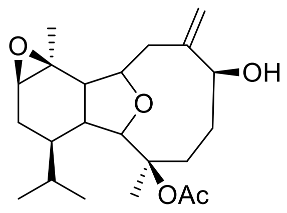

cladieunicellin C

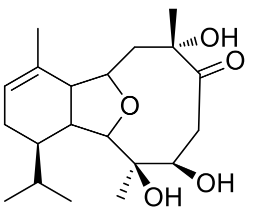

cladieunicellin D

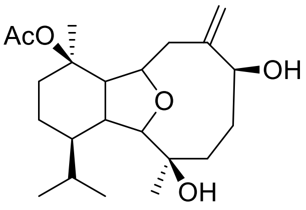

cladieunicellin G

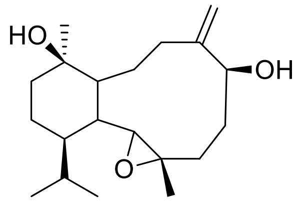

6-*epi*-cladieunicellin F

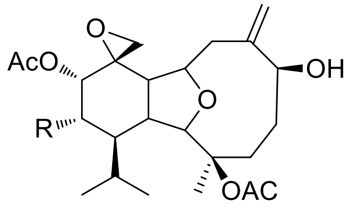

klymollin C R = OAc
klymollin D R = H

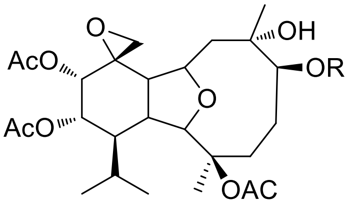

klymollin E R = Ac
klymollin F R = $OCO(CH_2)_{12}CH_3$
klymollin G R = $OCO(CH_2)_{14}CH_3$
klymollin H R = CHO

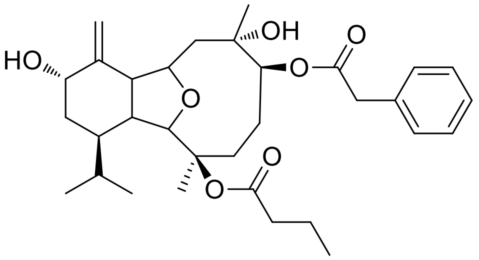

klymollin M

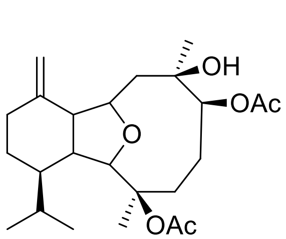

klymollin X

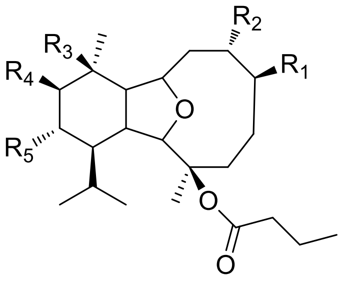

klysimplexin A $R_1$ = =O, $R_2$ = $CH_3$, $R_3$ = OAc, $R_4$ = H, $R_5$ = OH
klysimplexin B $R_1$ = =O, $R_2$ = C=$CH_2$, $R_3$ = OAc, $R_4$ = H, $R_5$ = OH
klysimplexin C $R_1$ = OH, $R_2$ = C=$CH_2$, $R_3$ = OAc, $R_4$ = H, $R_5$ = OH
klysimplexin L $R_1$ = OH, $R_2$ = C=$CH_2$, $R_3$ = OH, $R_4$ = $OCOCH_2CH_2CH_3$, $R_5$ = OAc
klysimplexin M $R_1$ = OOH, $R_2$ C=$CH_2$, $R_3$ = OH, $R_4$ = $OCOCH_2CH_2CH_3$, $R_5$ = OAc
klysimplexin N $R_1$ = OH, $R_2$ = C=$CH_2$, $R_3$ = OH, $R_4$ = OAC, $R_5$ = OAc

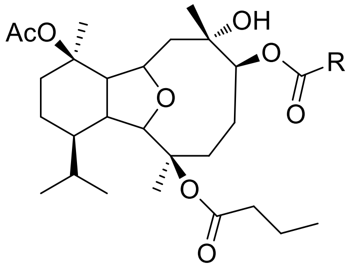

klysimplexin J R = $(CH_2)_{14}CH_3$
klysimplexin K R = $(CH_2)_{16}CH_3$

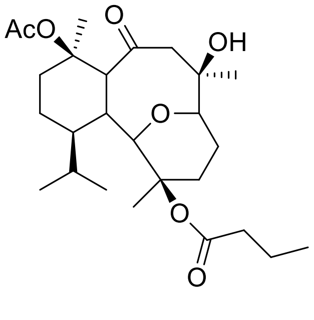

klysimplexin S

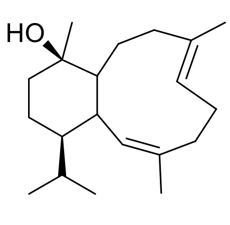

klysimplexin R

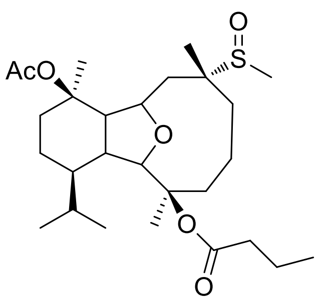

Klysimplexin sulfoxide A

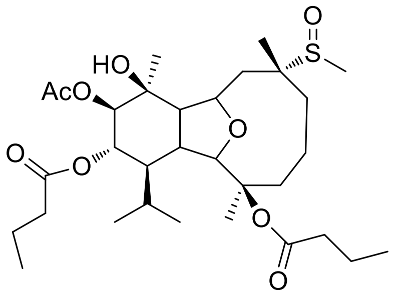

Klysimplexin sulfoxide B

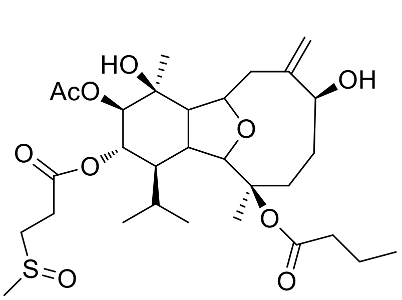

Klysimplexin sulfoxide C

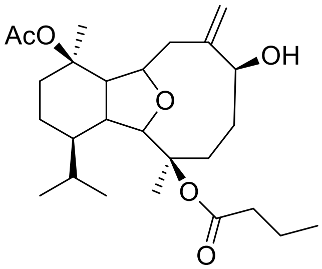

symplexin A

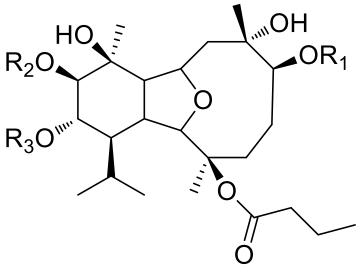

symplexin D $R_1$ = $CO(CH_2)_2CH_3$, $R_2$ = Ac, $R_3$ = $CO(CH_2)_2CH_3$
symplexin E $R_1$ = $COCH{=}CH_2$, $R_2$ = AC, $R_3$ = $CO(CH_2)_2CH_3$

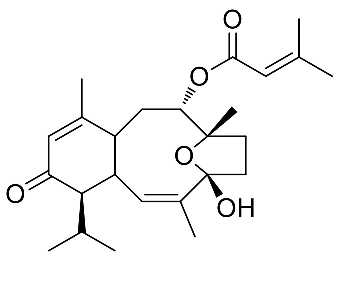

Valdivone A

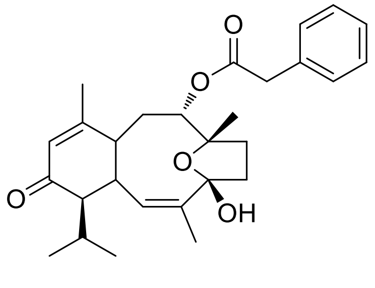

Valdivone B

# ANTI-INFLAMMATORY MARINE BRIARANE DITERPENOIDS

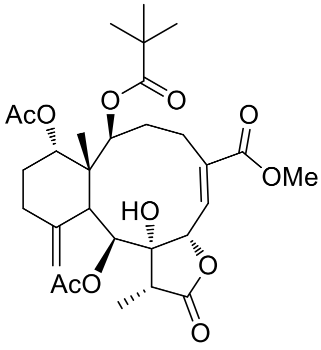

frajunolide P

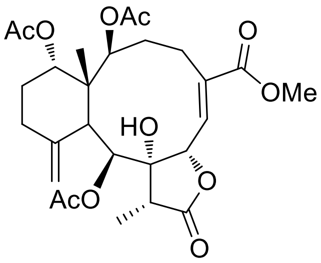

frajunolide Q

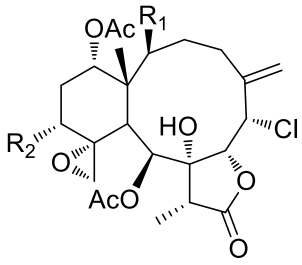

juncenolide H $R_1 = R_2 = OAc$

juncenolide I $R_1 = OCOCH(CH_3)_2$, $R_2 = OAc$

juncenolide J $R_1 = OAc$, $R_2 = OCOCH_2CH(CH_3)_2$

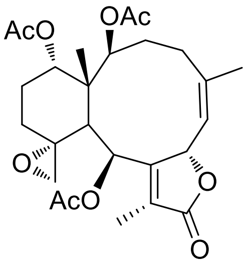

juncenolide K

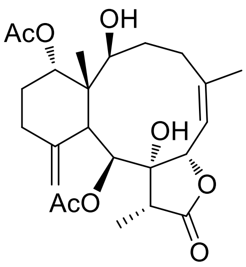

juncenolide N

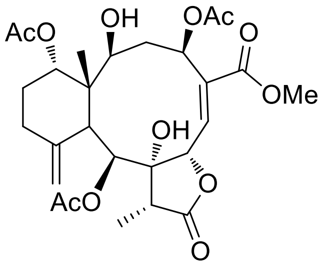

juncenolide O

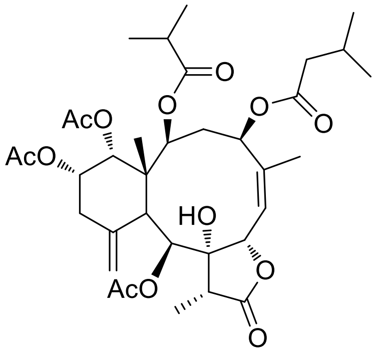

junceol E

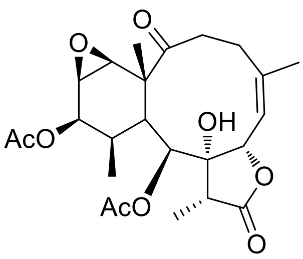


briarenolide E

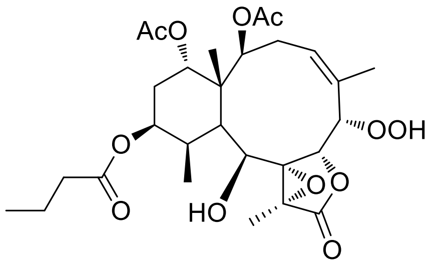


briarenolide F

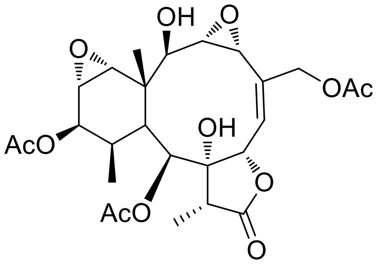


briarenolide K

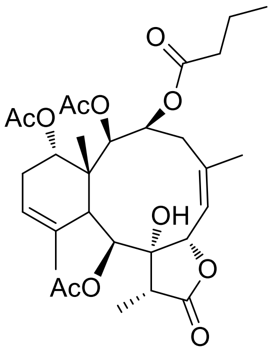


briarenolide L

# ANTI-INFLAMMATORY MARINE CEMBRANE DITERPENOIDS

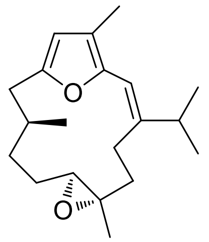


crassarine H

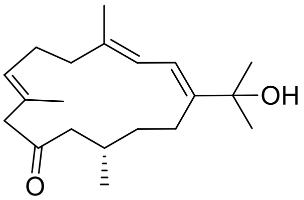


grandilobactin D

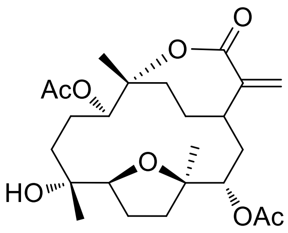


querciformolide C

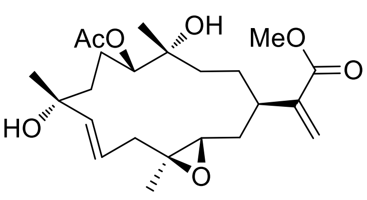


querciformolide E

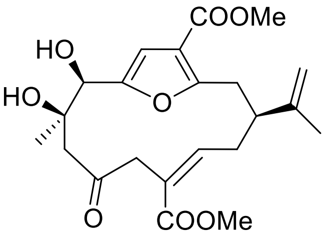


sinumaximol A

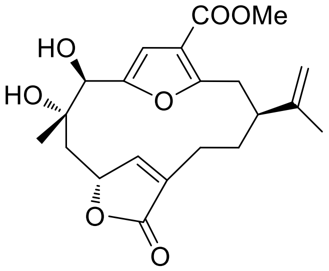


sinumaximol B

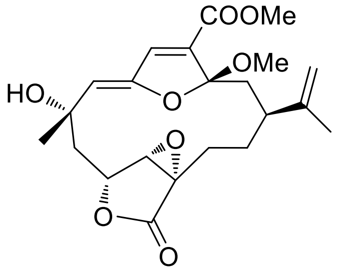


sinumaximol C

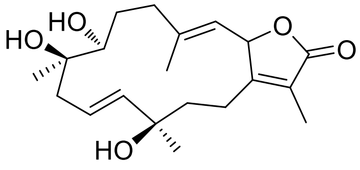


sinumaximol G

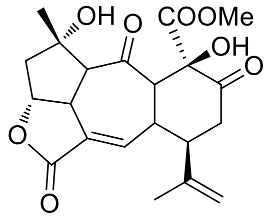


sinumaximol I

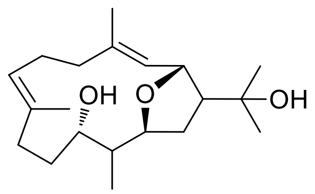


columnariol A

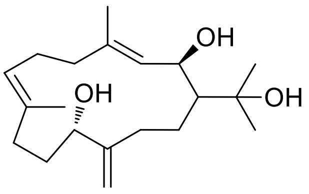


columnariol B

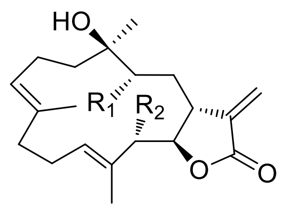


crassocolide A $R_1$ = OH, $R_2$ = OAc
crassocolide B $R_1$ = OAc, $R_2$ = OAc
crassocolide D $R_1$ = OH, $R_2$ = H

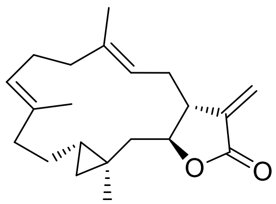


crassocolide E

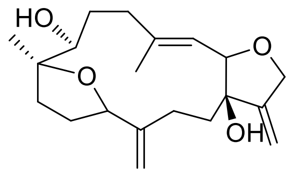


crassumol E

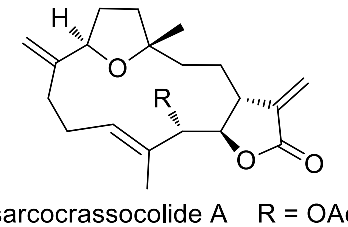

sarcocrassocolide A R = OAc
sarcocrassocolide C R = H

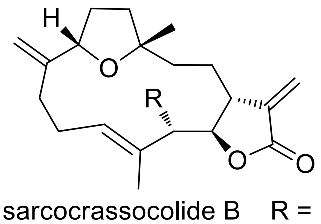

sarcocrassocolide B R = OAc
sarcocrassocolide D R = H

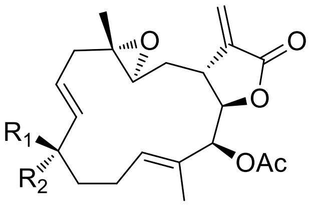

sarcocrassocolide F R = $CH_3$, $R_2$ = OOH
sarcocrassocolide G R = OOH, $R_2$ = $CH_3$
sarcocrassocolide H R = $CH_3$, $R_2$ = OH
sarcocrassocolide I R = OH, $R_2$ = $CH_3$

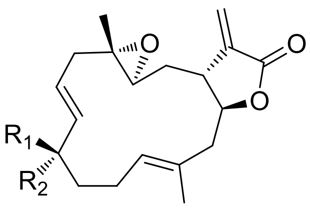

sarcocrassocolide J $R_1$ = $CH_3$, $R_2$ = OOH
sarcocrassocolide K $R_1$ = $CH_3$, $R_2$ = OH
sarcocrassocolide L $R_1$ = OH, $R_2$ = $CH_3$

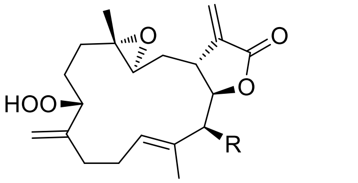

sarcocrassocolide M R = OAc
sarcocrassocolide O R = H

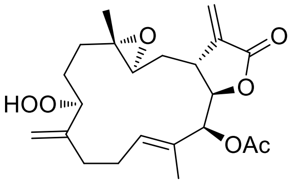

sarcocrassocolide N

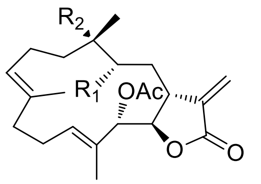

sarcocrassocolide P $R_1$ = OH, $R_2$ = OAc
sarcocrassocolide Q $R_1$ = OAc, $R_2$ = OH

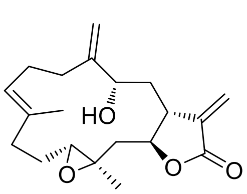

sarcocrassocolide R

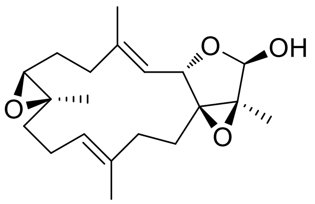

laevigatol A

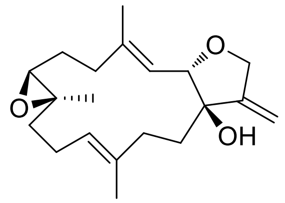

laevigatol B

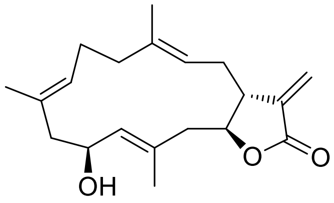

crassumolide A

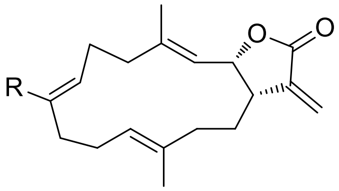

crassumolide B R = $CH_2OH$
crassumolide C R = COOMe

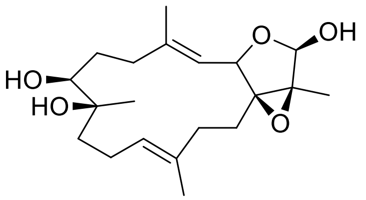

lobocrassol A

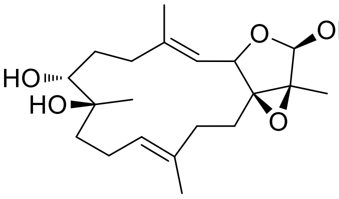

lobocrasol B

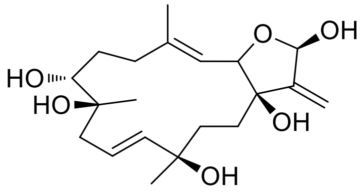

lobocrasol C

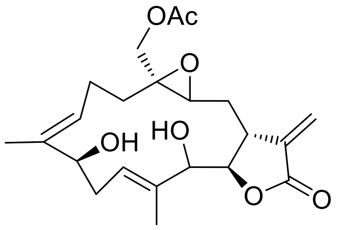

durumolide A

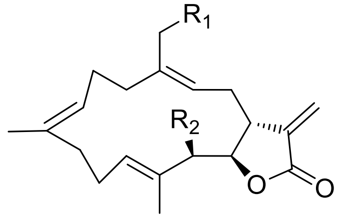

durumolide B $R_1$ = OAc, $R_2$ = OH
durumolide C $R_1$ = OH, $R_2$ = OH

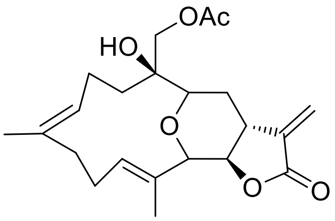

durumolide D

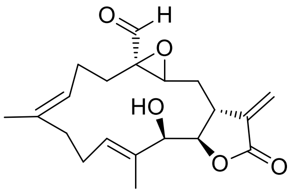

durumolide E

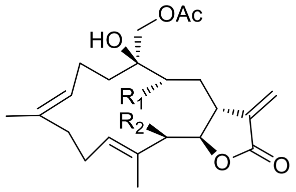

durumolide F $R_1$ = OH, $R_2$ = H
durumolide G $R_1$ = OAc, $R_2$ = OH
durumolide H $R_1$ = OAc, $R_2$ = OAc

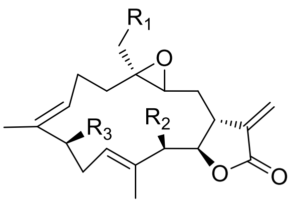

durumolide I $R_1$ = OAc, $R_2$ = OAc, $R_3$ = OH
durumolide J $R_1$ = $R_3$ = H, $R_2$ = OH

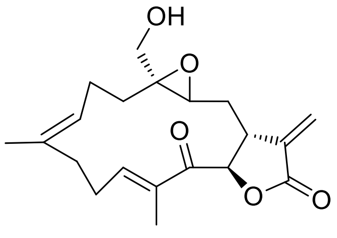

durumolide K

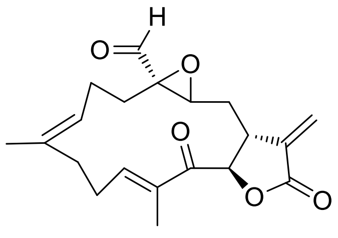

durumolide L

# ANTI-INFLAMMATORY DITERPENE GLYCOSIDES

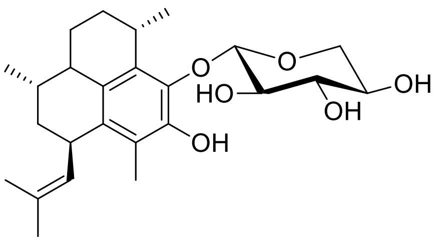


pseudopterosin A

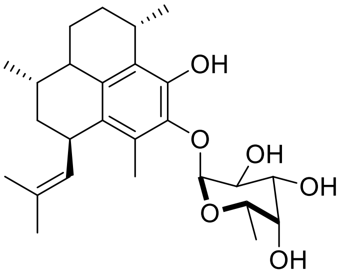


pseudopterosin E

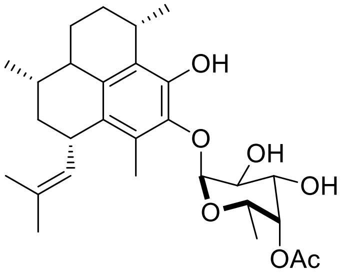


pseudopterosin Q

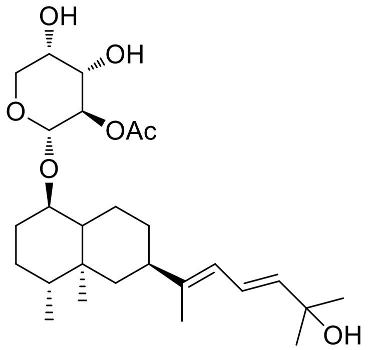


fuscoside A

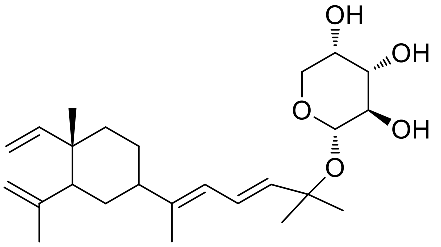


fuscoside B

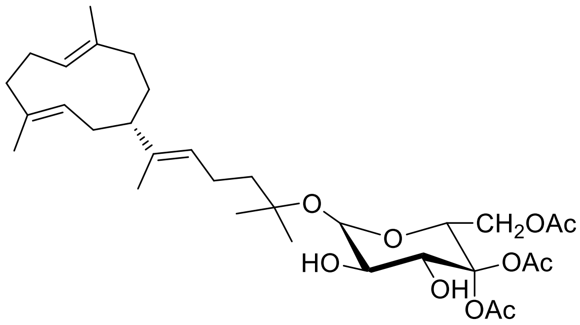


Calyculaglycoside B

# OTHER MARINE DITERPENES WITH ANTI-INFLAMMATORY ACTIVITY

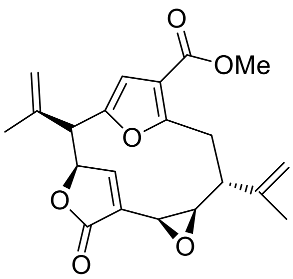
pseudopterolide

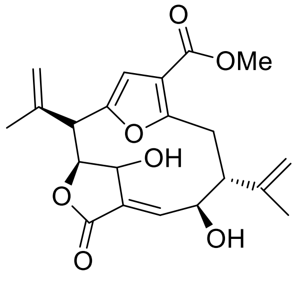
isogorgiacerodiol

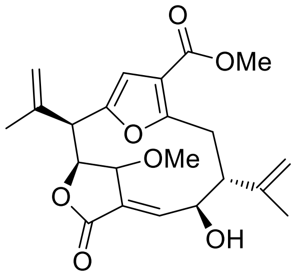
9-methoxy-isogorgiacerodiol

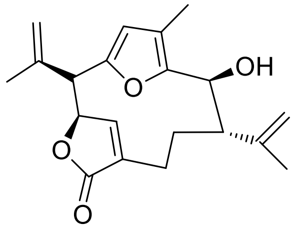
kallolide A

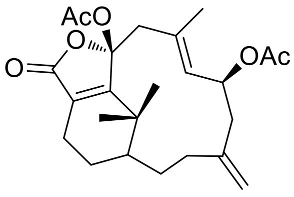
Cespitularin S

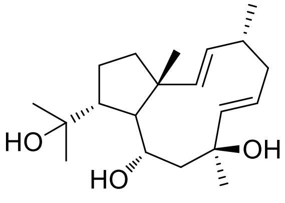
Dolabelladienetriol

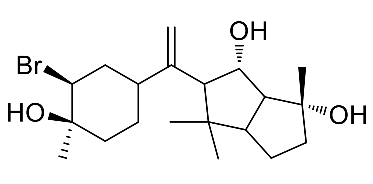
Neorogioltriol

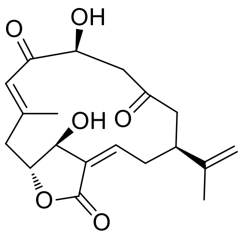
gyrosanolide A

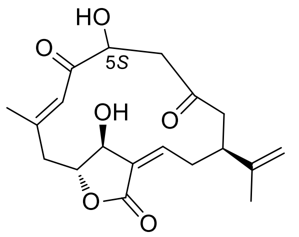
gyrosanolide B

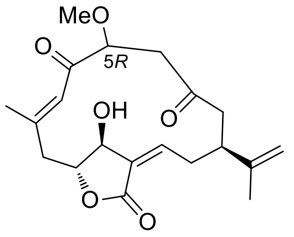
gyrosanolide C

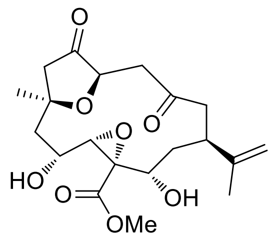
gyrosanin A

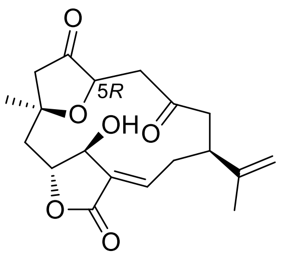

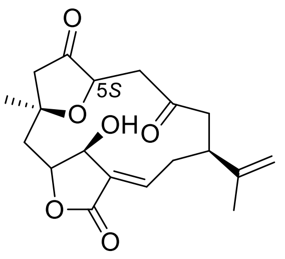

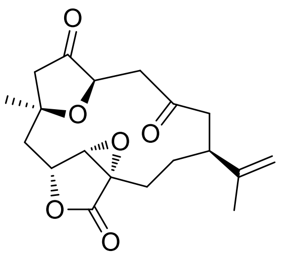

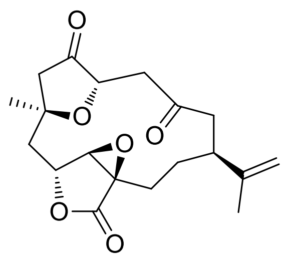

norditerpenoids from *Sinularia gyrosa*
